# Study of Piezoresistive Behavior of Smart Cement Filled with Graphene Oxide

**DOI:** 10.3390/nano11010206

**Published:** 2021-01-15

**Authors:** Rongxin Guo, Yuxia Suo, Haiting Xia, Yang Yang, Qianmin Ma, Feng Yan

**Affiliations:** 1 Yunnan Key Laboratory of Disaster Reduction in Civil Engineering, Faculty of Civil Engineering and Mechanics, Kunming University of Science and Technology, Kunming 650500, China; guorx@kust.edu.cn (R.G.); 20173110003@stu.kust.edu.cn (Y.S.); haiting.xia@kust.edu.cn (H.X.); 20140018@kust.edu.cn (Q.M.); yanfeng@kust.edu.cn (F.Y.); 2 Faculty of Civil Aviation and Aeronautics, Kunming University of Science and Technology, Kunming 650500, China

**Keywords:** carbon nanomaterials, mechanical properties, graphene oxide/cement composites, piezoresistive sensitivity, smart cement

## Abstract

A cement-based piezoelectric composite, modified by graphene oxide (GO), was prepared to study piezoresistive capacity. The testing confirms that GO is more effective than other carbon nanomaterials at improving piezoresistive sensitivity of cement-based composites, because the content of GO in cement paste was much lower than other carbon nanomaterials used in previously published research. Further investigation indicates that the addition of GO significantly improved the stability and repeatability for piezoresistive capacity of cement paste under cycle loads. Based on experiment results, the piezoresistive sensitivity of this composite depended on GO content, water-to-cement weight ratio (w/c) and water-loss rate, since the highest piezoresistive gauge factor value (GF = 35) was obtained when GO content was 0.05 wt.%, w/c was 0.35 and water-loss rate was 3%. Finally, microstructure analysis confirmed that conductivity and piezoresistivity were achieved through a tunneling effect and by contacting conduction that caused deformation of GO networks in the cement matrix.

## 1. Introduction

Currently, construction materials are developed to be multifunctional, including self-healing, self-sensing and durable. Structural health monitoring (SHM) technology has necessarily been developed since it is able to monitor and evaluate the safety and stability performances of engineered structures [1,2]. However, the drawbacks of traditional sensors (strain gauges, accelerometers, optical fiber sensors, etc.), including poor structural compatibility, low service life and increasing long-term maintenance cost, limit their application. Cement composites filled with conductive fillers such as carbon nanomaterials have attracted more attention because of their resistivity–strain sensitivity to external loadings, i.e., piezoresistivity [3]. Therefore, piezoresistive cement-based strain sensors (PCSS) may have industrial applications due to their good compatibility, high sensitivity and durability, and economical future maintenance expenses [4,5].

Normal carbon-based nanomaterials used in cement composites include carbon fiber (CF), carbon nanofiber (CNF), carbon black (CB), carbon nanotubes (CNTs) and graphene [6,7]. CF is one of the first materials used in PCSS, monitoring the health of concrete through the relationship between volume resistivity and compressive stress [8]. With advances in nanomaterials, more types of carbon nanomaterials are increasingly applied in PCSS [9,10], but the accuracy of self-sensing cement composites filled with CF and CNTs is controversial. The application of graphene in composites is due to its large surface area, high mechanical strength and high electrical conductivity [11,12,13]. Cement composites modified by graphene nanoplatelets (GNP) present with pressure sensitivity, with the gauge factor reaching a peak value of 7.783 [14]. However, the common defect of graphene and other carbon nanomaterials is hydrophobicity in aqueous solutions caused by strong van der Waals forces between nanosheets, resulting in poor dispersion in the cement matrix and weakening the ultimate performance of graphene [15]. According to previous studies, graphene oxide (GO) is the most popular derivative of graphene used in cement composites with a satisfactory hydrophilic property [16]. The molecular composition of GO contains some functional groups in its base structure, such as hydroxyls (-OH), carboxyls (-COOH), carbonyls (-C=O-) and epoxides (-CH(O)CH-), which cause a strong interface effect with cement and an ideal dispersion in the matrix [17,18]. Earlier studies focused on the mechanical properties of cement composites filled with GO [19]. Usually, GO shows more obvious advantages for improving the compressive strength of cement composites at lower concentrations than other carbon nanomaterials [20]. For example, a nearly 48% enhancement in compressive strength is achieved by incorporating only 0.05 wt.% GO in the cement matrix compared to that without GO [21]. However, research on piezoresistive capacities of cement comprising GO is still rare, despite the publication of a few studies on electrical properties [22]. Moreover, piezoresistivity is quite sensitive to water content in the cement matrix [23,24]. Therefore, water-to-cement ratio (w/c) and moisture contents must be considered.

In this paper, graphene oxide/cement composites (GO/CC) with piezoresistive sensitivity were prepared with different w/c ratios. The workability and compressive strength of composites with different GO contents were tested. Then, the electrical and piezoresistive properties of GO/CC with different contents of GO and moisture were investigated. Finally, the mechanisms of conduction and piezoresistivity of GO/CC were analyzed to study the feasibility of industrial applications of GO/CC strain sensors.

## 2. Materials and Methods

### 2.1. Raw Materials

Portland cement type 42.5R (Huaxin Cement Co., Ltd., Kunming, China) was used to prepare the cement paste. The chemical composition of cement obtained by X-ray Fluorescence (XRF) is listed in Table 1. GO gel with 10 mg/mL was procured from Chengdu Organic Chemicals Co. Ltd., Chengdu, China, and its chemical and physical properties provided by the supplier are given in Table 2. The electrical conductivity of GO sheets is approximately 13 S/m, which was estimated by the method used in [25]. Polycarboxylate superplasticizer (PCs) obtained from Sika Building Material Ltd., Chengdu, China, was used as a surfactant to promote the dispersion of GO in the cement paste.

### 2.2. Preparation of Cement Paste

The GO gel was firstly mixed with 0.2 wt.% PC solution under ultra-sonication for 30 min to ensure homogeneous dispersion [26]. This aqueous suspension was then mixed with cement in a mixer (NJ-160B, Wuxi, China). The water-to-cement weight ratio (w/c) varied from 0.3 to 0.4, and the concentrations of GO were 0.05, 0.1, 0.15 and 0.2%, respectively. By contrast, plain cement paste without GO was prepared as the control specimen. Specimens with a size of 25 mm^3^ were made for electrical testing. A pair of copper electrodes (20 × 35 mm) were embedded in the cement paste. The distance between the electrode and the edge of the specimen was kept at 5 mm for all samples. The mixing, casting, demolding and curing processes for all specimens were similar to those in previous works [27]. To avoid effects caused by moisture and polarization, all specimens were dried for 27 h before testing.

### 2.3. Test for Electrical Resistivities

A constant direct voltage (U) of 10 V was applied to the two probes by a power supply (DPS-305CF), and the current difference (I) was measured by a digital multimeter (Agilent 34410A, Keysight, Santa Rosa, American) connected to the copper electrodes. The electrical resistance (R) of the sample was calculated by Ohm’s law, i.e., R = U/I. Considering that polarization is easily caused by ions (Ca^2+^, OH^−^ etc.) in pore solution of cement-based composite under a direct current (DC) electric field, all specimens were first dried in an oven at 40 °C until weights were constant. The dried samples were then electrified for a certain amount of time in order to further reduce the polarization effect before electrical tests [11,28].

It is known that the four-probe method is more acceptable than the two-probe method when considering the influence of contact resistance. However, the contact resistance of a specimen with a small size (25 mm^3^) was much smaller than the measured resistance, which had little influence on the test results [29]. Therefore, the two-electrode method is more appropriate here since it is simple to manipulate and the test results are more accurate [30].

To consider the effect of nature and geometry of samples, the electrical resistance of the composites was converted into the bulk electrical resistivity *ρ*, which was calculated by:

*ρ* = RA/L, (1)

where L is the length between electrodes and A is the cross-sectional area between the copper electrodes and cement paste. All measurements were conducted at room temperature.

### 2.4. Test for Piezoresistive Capabilities

**Figure 1:**
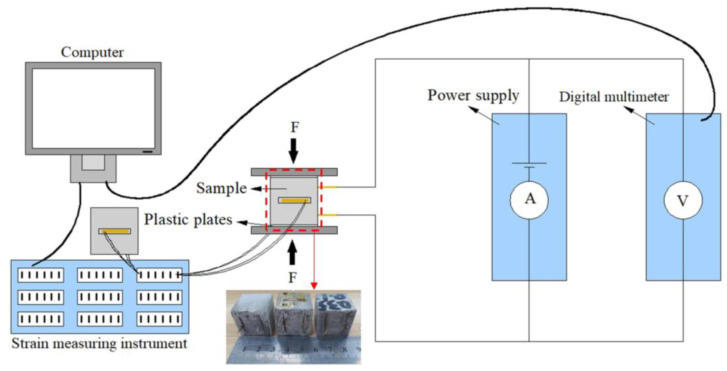
Schematic illustration of the piezoresistive properties test.

The piezoresistivity of the composites was analyzed by applying cyclic uniaxial compressive loads and simultaneously measuring the deformation and electrical resistance. A schematic diagram of this testing is shown in Figure 1. The cycle load was applied by a universal testing machine (CSS) with a loading rate of 0.5 kN/s. The minimum and maximum cycle loads were 0.1 and 6 kN, respectively, to ensure that the sample remained in the elastic regime during the testing. A preload of 0.1 kN was applied before the cycle load to maintain good contact between the specimen and the testing machine. Two plastic plates were placed on both ends of the specimen to prevent any unwanted electrical conduction. The electrical resistance of GO/CC during the cyclic loading was measured by the same method as above in Section 2.3. At the same time, the axial stain was measured by 10 mm strain gauges, with a temperature compensated, full bridge configuration, and the value was continuously collected via a strain measuring instrument (TST3826F-H). The piezoresistive sensitivity is usually evaluated by the gauge factor (GF), which can be defined as the fractional change of resistance (FCR) per strain unit. These are calculated according to [31]:

FCR = ΔR/R_0_, (2)

GF = FCR/ε,(3)

where ΔR is the change in electrical resistance, R_0_ is the initial electrical resistance and ε is the strain.

## 3. Results

### 3.1. Effect of GO Concentration on the Electrical Resistivities of GO/CC with Different Moisture Contents

According to previous compressive strength test results [27], samples with w/c = 0.35 feature the best compressive strength, which increases by 24% compared to cement paste without GO. Thus, they were selected for the electrical resistivity test with different moisture contents when GO concentrations change from 0.05 to 0.2 wt.%. The water loss rate (WL) indirectly indicates the moisture of cement matrix for all specimens, and it is calculated as follows:

WL = [(w_1_ − w_2_)/w_1_] × 100% (4)

where w_1_ is the weight of the specimen taken immediately out of the curing environment in water, and w_2_ is the weight of the specimen after a drying process in an oven at 50 °C. The corresponding WL for specimens dried for 0, 3, 9 and 27 h are 0, 3.0, 4.5 and 6.5%, respectively.

**Figure 2:**
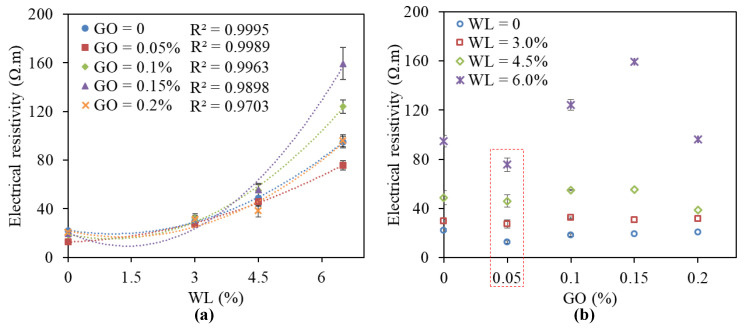
Electrical resistivity of the control and graphene oxide/cement composites (GO/CC) under (**a**) different rates of water loss and (**b**) different GO concentrations.

Figure 2 shows the effect of the moisture and the GO concentration on the bulk electrical resistivity of GO/CC. It can be observed from Figure 2a that with the increased water loss rate, the electrical resistivities for all specimens increase. The relationship between electrical resistivities and moisture was analyzed according to the fitted data using a least squares polynomial fit method. As shown in Figure 2a, the coefficients of determination (R^2^) were quite close to 1, reflecting a high-fitting degree of curves. It shows a quadratic polynomial correlation between electrical resistivities and moisture. This result directly indicated that the GO/CC electrical resistivity changes with the decreasing moisture in the cement matrix. In addition, the values of electrical resistivity at different GO concentrations are similar when measured at a low moisture condition, which shows that water plays the main role here. However, when the moisture content of the cement matrix was low (as WL = 4.5 and 6.5%), the electrical resistivity at different GO concentrations varied greatly, especially for the specimens under WL = 6.5%, which means GO plays a main role in conductivity.

Figure 2b further illustrates the effect caused by GO sheets in the cement matrix, since the specimens showed a decreased electrical resistivity when 0.05 wt.% GO was added. Such results can be explained in two aspects. Firstly, GO regulates the hydration product to form a compact microstructure and increases the intrinsic conductivity of the cement matrix through ionic conduction. According to our previous research [27], the microstructure of cement with appropriate GO concentration (i.e., 0.05 wt.%) indicates a more compact and regular microstructure since the cracks and holes decrease. As a result, a conductive path in cement is easy to form and resistivity decreases. For cement without GO or with a high content of GO (i.e., 0.15 wt.%), loose and irregular microstructures are observed [27], which destroy the conductive path and increase resistivity, as shown in Figure 2b. Secondly, well-dispersed GO plays a certain role in decreasing electrical resistivity, because conductivity of the GO sheet (13 S/m) is much higher than that of the cement matrix (0.0105–0.0451 S/m) and deionized water (0.0004 S/m). For the specimens with concentrations over 0.05 wt.%, their resistivities are close to or higher than the control specimen. Poor GO dispersion and aggregation at high content negatively influence the improvement of electrical conductivities. Thus, it can be inferred that water plays the main role in electrical properties at high moisture content [22,32], but the effect of GO sheets on GO/CC resistivities could not be neglected at low moisture content.

### 3.2. Effect of GO Concentration on the Piezoresistive Capacities of Cement Paste

**Figure 3:**
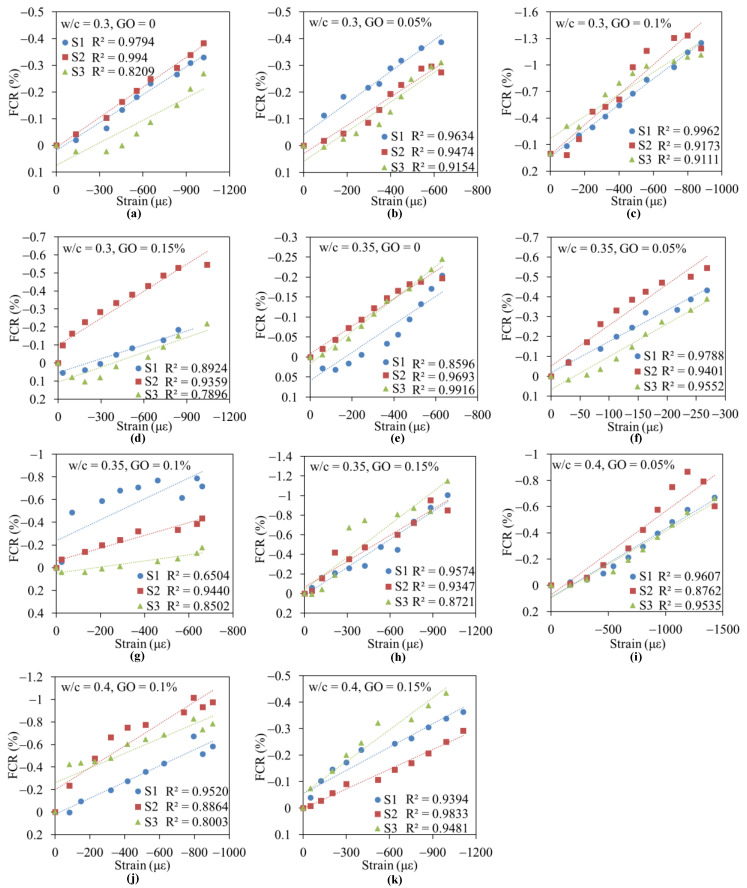
Corresponding changes in resistance of GO/CC under different strains for specimens at different w/c: (**a**) w/c = 0.3, GO = 0; (**b**) w/c = 0.3, GO = 0.05%; (**c**) w/c = 0.3, GO = 0.1%; (**d**) w/c = 0.3, GO = 0.15%; (**e**) w/c = 0.35, GO = 0; (**f**) w/c = 0.35, GO = 0.05%; (**g**) w/c = 0.35, GO = 0.1%; (**h**) w/c = 0.35, GO = 0.15%; (**i**) w/c = 0.4, GO = 0.05%; (**j**) w/c = 0.4, GO = 0.1%; (**k**) w/c = 0.4, GO = 0.15%.

The specimens were dried in an oven at 40 °C for 27 h before the piezoresistive capacities tests to eliminate the influence of moisture. The values of FCR via compressive strain of GO/CC with different w/c are illustrated in Figure 3. The slopes of these linear fitting curves represent the GFs, and their values were listed in Table 3. Three specimens of each GO concentration at different w/c were analyzed in the piezoresistive capacities test, and they were labeled, respectively, as S1, S2 and S3. The plain cement specimen when w/c = 4 shows no piezoresistive responses, so its fitted results was not shown in the figure. The experimental results show that the curves of cement paste filled with GO have a larger slope than the one without GO, which means the corresponding GF value is higher. Combined with Table 4, it can be found that the specimens with 0.1 wt.% GO at the condition of w/c = 0.3 and w/c = 0.4 induce 275 and 800% increments for GF, respectively, when compared to the plain sample. The maximum increment of GF is obtained when wt.% GO is 0.05 and w/c = 0.35 (GF = 16), which is 300% higher than the corresponding value of a plain sample. We can further find in Figure 3 that the slope of FCR–strain (ε) curves first increases, and then decreases when GO content changes from 0 to 0.15 wt.%. Thus, the piezoresistive capacity of a cement composite is effectively enhanced by GO when w/c and GO content represent appropriate values.

Table 4 lists different GF values of for the composites modified by GO, CNTs, CNF and GNP [14,31,33], respectively. It is shown that the GF values of composites containing CNF and graphene are both lower than the ones containing GO. Meanwhile, the corresponding amounts of CNF and graphene are far more (1 and 2 wt.%) than that of GO (0.05 wt.%), when the GF values are similar. Therefore, GO is an effective and appropriate choice for the preparation of a smart concrete sensor when compared to other carbon nanomaterials. More importantly, a small content of GO (0.05 wt.%) can simultaneously improve compressive strength and the gauge factor. Thus, it is meaningful and efficient for GO to solve the imbalance problem between mechanical strength and electrical sensitivity for nanocomposites.

### 3.3. Effect of GO Concentration on the Dynamic Piezoresistive Response of Cement Paste

**Figure 4:**
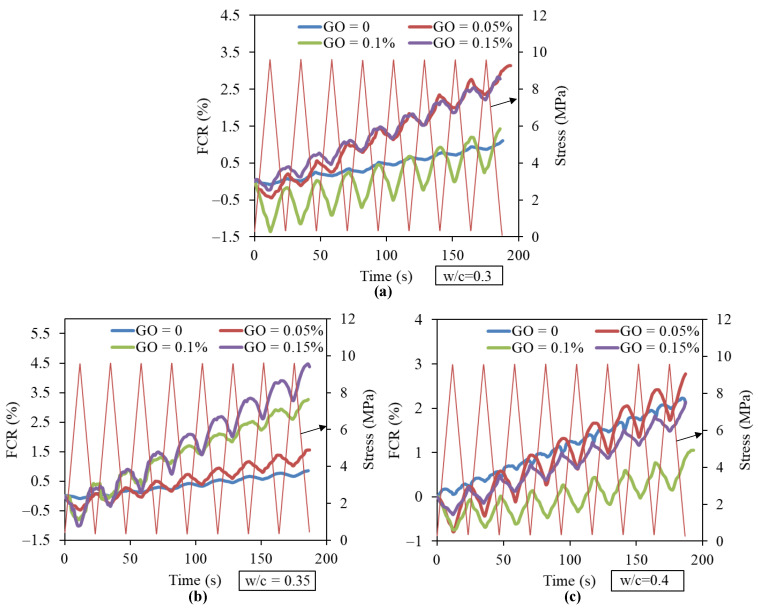
Corresponding fractional changes in resistance under compressive stress for specimens at different w/c: (**a**) w/c = 0.3; (**b**) w/c = 0.35; and (**c**) w/c = 0.4.

In order to promote the practical application of this GO/CC composite in monitoring structure health, FCR values of the composite under cycle stress were tested. Figure 4 plots the relationship of cycle compressive stress via FCR for cement paste with different GO concentrations (0, 0.05, 0.1 and 0.15 wt.%) and w/c. The specimen with 0.2 wt.% GO is not selected due to its worst fluidity. Liu et al. [22] suggested that GO might not be a good candidate to prepare smart concrete because GO provided almost no contribution to the electrical conductivity of concrete. However, it is interesting to note that the addition of GO significantly improves the piezoresistive sensitivity of cement paste under cycle stress load in this study. It can be seen from Figure 4 that the plain specimens without GO also indicate weak piezoresistive responses since the electrical resistivity expressed in Equation (1) changes with the deformation of the specimen. However, the cement paste filled with GO indicates synchronous and negative responses with variations of cycle loads. The maximum amplitudes of FCR are 1.4% (0.1 wt.% GO), 1.0% (0.15 wt.% GO-0.35) and 0.8% (0.05 wt.% GO) when w/c takes 0.3, 0.35 and 0.4, respectively. Furthermore, FCR value becomes larger when the times of the loading/unloading cycle keep increasing, since the bonding effect between GO and the cement matrix is gradually weakened during this process [34], and damage or cracks in the cement matrix induced by cycle loading also increase the value of FCR [24].

### 3.4. Effect of Moisture Content on the Piezoresistive Capacity of Cement Paste

**Figure 5:**
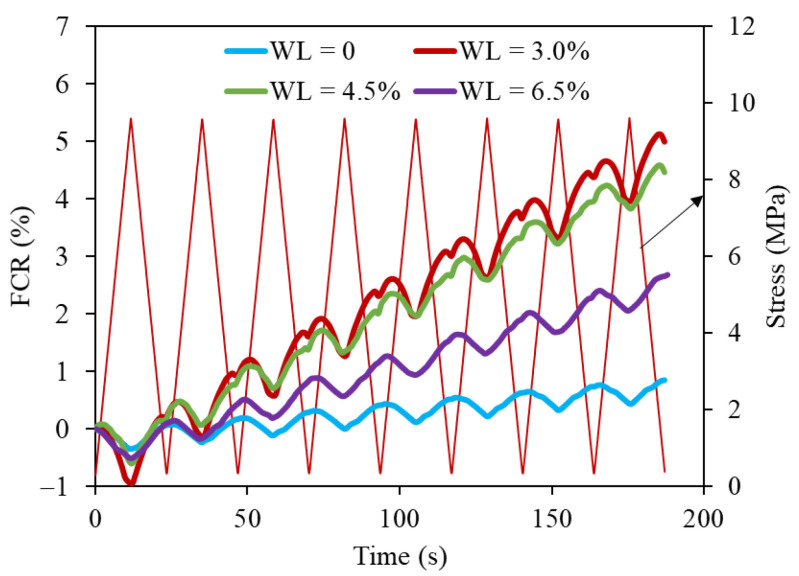
Corresponding changes in resistance under compressive stress of 0.05GO-0.35 with different moisture contents.

Figure 5 shows the piezoresistive responses of 0.05GO-0.35 with different moisture contents under cycle loading when the stress amplitude is 9.6 MPa. The results show that FCR values change with the cyclic loading synchronously, exhibiting good stability and repeatability, even though the relationship between FCR and compressive stress is not completely reversible (as mentioned in Section 3.3). The change in moisture content has almost no influence on the amplitude of FCR. Therefore, all GO/CC specimens with different moisture contents indicate piezoresistive ability.

**Figure 6:**
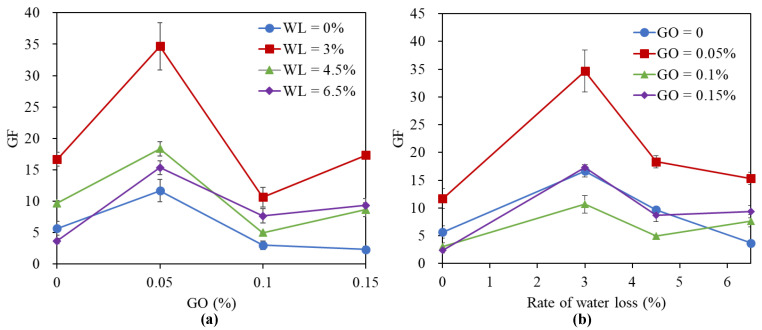
GF values of the control and GO/CC when WL = 0, 3.0%, 4.5% and 6.5% with (**a**) different concentrations of GO and (**b**) different water cement ratios.

Figure 6 shows the piezoresistivity of GO/CC under different water loss rates and GO contents. As shown in Figure 6a, the cement paste containing 0.05 wt.% GO can greatly enhance the value of GF when compared to the sample without GO at all water loss rates. Figure 6b shows that the correlation between GF and the water loss rate is not linear because GF increases first and then decreases with decreasing water content. The results in Figure 6a,b show that *GF* can approach a maximum value when the concentration of GO and the water loss rate reach an appropriate value. The optimal water loss rate is 3.0% and the optimal GO content is 0.05 wt.% when the specimen in this condition provides a much higher GF value (GF = 35) than other ones.

## 4. Discussion

Based on the above results, the conductive and piezoresistive mechanism of GO in cement paste can be further discussed here. GO/CC is a porous system that consists of GO sheets, hydration products, non-hydrated cement particles and pores. According to Wen and D.D.L [35], electrical conduction in carbon-based cement composites is induced by both electronic conduction and ionic conduction. For the specimens filled without GO, the electrical conduction of the cement matrix depends on the ionic conduction caused by the movements of ions (Ca^2+^, Na^+^, etc.) in porous solutions. For GO/CC, conductive ways are combined by the electronic conduction on GO sheets and the ionic conduction of cement matrix between GO sheets. Electrons can jump among the adjacent GO sheets (tunneling effect) or they can flow through the connected GO sheets (contacting conduction).

The dominant conductive way in GO/CC is different when the moisture in the cement matrix changes. Based on different amounts of water content in the matrix, the moisture state of cement composite consists of a dry stage, low-water stage, medium-water stage and high-water stage [36]. In the high-water stage (WL = 0), ionic conduction dominates the electrical conductivity. The current can pass through the porous solution with low resistance, presenting a low electrical resistivity. However, the water layer can block the motion of electrons between different GO sheets, which decreases the electronic conduction. At medium-water and low-water stages, the ionic conduction is weakened, and electrical resistivity keeps increasing. In the dry stage (WL = 6.5%), the ionic conduction path is disconnected since the conductivity of GO/CC is only caused by tunneling effect and contacting conduction of GO sheets. Thus, the electrical resistivity of GO/CC is obviously lower than CC without GO. In addition, the functional groups such as -OH or -COOH on the GO surface may enhance the interaction with ions to improve ionic conduction [37]. As mentioned above, well dispersed GO can regulate the formation of a compact microstructure, which may prevent the evaporation of residual water in the matrices and enhance ionic conduction, whereas poor dispersion of GO leads to agglomerates that destroy the formation of a conductive network.

For electronic conduction, contacting conduction and tunneling conduction both play a role, but the tunneling effect among GO sheets is the key factor causing the piezoresistive property [38]. The above experimental results show that the piezoresistive capability for the cement paste without GO is weak since only an ion solution in pores is effective, but it is not sensitive to the deformation of the cement composite under compression [39]. After the addition of GO, tunneling and contacting conductions are caused. In this case, decreasing the distance between GO sheets under compressing loads leads to a reduction of the tunneling distance and an increase in contact between GO sheets [40]. As a result, the motion of electrons is promoted and electrical resistivity decreases under compressing loads, which indicates a synchronous piezoresistive response.

The difference of piezoresistive properties GO/CC with different GO concentrations is closely related to GO dispersibility in the cement matrix. Shorter distances among uniformly dispersed GO sheets construct more tunneling conductive paths (an electrical current can be transferred though the tunneling effect when the distance of tunneling is within 1.8 nm [41]). The piezoresistive capability of cement paste with appropriate GO content reaches the best piezoresistive sensitivity due to the intense tunneling effect caused by the best dispersed GO sheets. As a result, GF values of GO/CC increase by 275% (0.1 wt.% GO, GF = 15), 300% (0.05 wt.% GO, GF = 16) and 800% (0.1 wt.% GO, GF = 8) compared to the cement paste without GO when w/c takes 0.3, 0.35 and 0.4, respectively. It is also inferred that adding GO into cement is an effective way to improve the piezoresistive capacity of cement composites

The water content in the cement matrix induces a high effect on the piezoresistivity of GO/CC [42]. With the increase of water loss rate in the cement matrix, the GO/CC piezoresistivity first increases and then decreases. The piezoresistive sensitivity of GO/CC decreases when the water content in cement matrix reaches a high-water level (WL = 0), because the strong ionic conduction in the matrix plays a major role and is insensitive to deformation. By reducing the water in the cement matrix, the ionic conduction decreases but the tunneling effect is enhanced. The best piezoresistive sensitivity is obtained when WL takes an appropriate value (as shown in Figure 6), because the tunneling effect is the most effective at this time. However, when the water content in the cement matrix is low (WL >4.5%), the poorer electrical conductivity of the cement matrix makes it harder to form a conductive network and presents low piezoresistive sensitivity again.

## 5. Conclusions

In summary, the electrical resistivity and piezoresistive response of GO/CC were investigated. The mechanisms of the conductivity and piezoresistivity under different GO concentrations and moisture levels were analyzed. Based on research results, the following conclusions can be obtained:

(1) A conductive network with tunneling effect and contacting conduction is effectively formed by GO sheets in the cement matrix. Under cyclic loading, the distances between adjacent GO sheets change together with the variations of strain load and thus lead to synchronous piezoresistive responses.
(2) Adding GO to cement paste enhances conductive and piezoresistive properties, reaching the minimum electrical resistivity (*ρ* = 76 Ω·m at WL = 6.5%) and the maximum piezoresistive sensitivity (GF = 16) when GO content is 0.05 wt.% and w/c = 0.35.
(3) Compared with other carbon nanomaterials, GO is more efficient at improving the piezoresistive sensitivity of the composite since the GF value of GO/CC is similar with those of other carbon nano/cement composites when the content of GO is only 0.05 wt.%.
(4) When the water loss rate was 3%, the specimens filled with 0.05 wt.% GO at w/c = 0.35 showed highest piezoresistive sensitivity (GF = 35), since this appropriate water content in GO/CC promotes the formation of a conductive network with a tunneling effect the most and leads to high piezoresistivity, when more and less water content reduce the piezoresistive sensitivity.

## Figures and Tables

**Table 1:** Chemical composition of cement.

Component	wt.%
Calcium oxide (CaO)	52.61
Silicon dioxide (SiO_2_)	22.04
Aluminium oxide (Al_2_O_3_)	7.97
Ferric oxide (Fe_2_O_3_)	4.17
Sulfur trioxide (SO_3_)	3.29
Magnesium oxide (MgO)	2.41
Potassium oxide (K_2_O)	0.90
Sodium oxide (Na_2_O)	0.16

**Table 2:** The properties of graphene oxide (GO) used in this study.

Property	Value
Diameter (μm)	0.5–3
Thickness (nm)	0.55–1.2
Layers	<3
Purity (%)	>99
Color	Brown
Carbon (%)	68.44
Oxygen (%)	30.92
Sulfur (%)	0.63

**Table 3:** Gauge factor (GF) values of GO/CC at different w/c.

GO (wt. %)	GF		
	w/c = 0.3	w/c = 0.35	w/c = 0.4
0	4 (±0.44)	4 (±0.44)	0
0.05	6 (±0.89)	16 (±1.11)	5 (±0.44)
0.1	15 (±1.78)	6 (±2.00)	8 (±1.33)
0.15	4 (±0.89)	9 (±1.11)	3 (±0.67)

**Table 4:** Comparison of GF values for different piezoresistive cement-based strain sensors (PCSS).

Cement Composites	Content of Carbon Nanomaterials (wt.%)	GF	Increment of Compressive Strength When Compared to Control
GO/CC	0.05	16	Increase by 24%
CNTs/CC [33]	0.25	16	Increase by 7%
CNF/CC [31]	1	10	No significant variations.
GNP/CC [14]	2	8	Weak by 20%

## Data Availability

The data presented in this study are available on request from the corresponding author.
